# Analysis on curve negotiating ability of rail grinder in grinding state

**DOI:** 10.1038/s41598-022-13712-1

**Published:** 2022-07-08

**Authors:** Luqing Zeng, Dabin Cui, Yaodong Fu, Li Li, Zhanghong Liu

**Affiliations:** School of Mechanical Engineering, Southwest Jiaotong University, North 1st section, 2nd Ring Road, Chengdu City, 610031 China

**Keywords:** Electrical and electronic engineering, Mechanical engineering

## Abstract

Rail grinding becomes an important maintenance means of railway. Dynamic behavior of rail grinder is due to vehicle-track coupling relationship based on mechanical-electric-hydraulic coupling. Curve negotiating ability of rail grinder through modeling and simulation based on one-side grinding is studied in this paper. Simulation result is shown below. In typical case, rail grinding will increase transverse displacement of wheelsets, derailment coefficient of wheels in front wheelset, and unloading rate of wheelsets. In other case, increase of rail irregularity amplitude and decrease of its wavelength, which aggravates fluctuation of grinding power, has little influence on curve negotiating ability. When line radius curvature decreases, compared to state without grinding, decline to curve negotiating ability of state with grinding is more significantly. When number of grinding wheels at work increases, lateral displacement of wheelset, derailment coefficient of wheels in front wheelset, and unloading ratio of wheelsets increase. In short, rail grinding will significantly deteriorate curve negotiating ability of rail grinder.

## Introduction

Rail grinding becomes a common method of rail maintenance around the world^1–3^. With expansion of urban metro network, traffic volume increases exponentially. This brings great challenges to rail grinding and promotes development and utilization of precise grinding technology and related equipment for metro lines^4–7^. Under development of vehicle dynamics theory for many years, relevant theoretical research models went through from simple wheel model to wheelset-bogie-vehicle model, train formation model and vehicle-track (or under-track foundation) coupling model. Zhai-Sun model^8,9^ is the representative. Rail grinding can improve vehicle dynamic behavior^10–12^. Unfortunately, due to limit of the involved field and application scope, scholars focus on wheel track relationship rather than grinding force. Therefore, dynamic behavior of rail grinder in grinding process is nearly a blank overseas. Grinding process is a dynamic grinding based on travelling process, so grinding power and dynamic behavior influence each other. If rail grinder itself is unstable, it may have a poor impact on grinding effect. In extreme cases, it may lead to increasement of rail irregularity in some operation sections ^13^. And curve negotiating capacity is an important part of dynamic behavior in rail grinder.

Wang^14^ established a multi-rigid body dynamic model of PGM-48 rail grinder by using SIMPACK, and analyzed the influence of buggy primary stiffness on its dynamic behavior. Zhang^15^ established dynamic model of GMC-96X rail grinder by using SIMPACK. Grinding force between grinding wheel and rail as well as pressure fluctuation of grinding hydraulic system are considered in the model. Influence of hydraulic system itself and influence of grinding car on buggy are not considered. So dynamic coupling relationship between rail grinder (including rail grinding car, buggy, hydraulic system, grinding wheel) and rail can not be realized in his model. Nie^16^ used AMESim software to establish pressure output system of a single grinding wheel in the field of rail grinder for normal-speed line, and put forward suggestions on how to reduce pressure fluctuation for air pressure system. Tang^17^ simulated pressure control of three-way proportional pressure reducing valve, and simulated influence of grinding pressure output affected by rail corrugation. Zhi^18^ established coupling model between rail grinder and grinding behavior, and analyzed the influence of buggy's lateral movement on vertical and lateral displacement of grinding wheel in grinding process under background of rail grinder used in existing line. Influence of hydraulic system is not considered in this model. Fan^19^ established dynamic model of abrasive belt grinding, and analyzed feasibility of high-speed abrasive belt grinding by studying dynamic behavior under straight and curved lines. However, influence of hydraulic system wasn't considered, too.

In this paper, vehicle-track coupling of rail grinder is established on the coupling of mechanical, hydraulic and control system. Curve negotiating ability of rail grinder under real-time interaction of the above three systems are studied. It can comprehensively reflect the impact of whole grinding system on curve negotiating ability of rail grinder.

## Operation principle

Rail grinding is based on constant travelling speed of rail grinder. For positive grinding, grinding speed is generally lower than 20 km/h. Grinding is divided into pre-grinding and corrective grinding. Its purpose is to make rail profile meet requirement or reduce rail irregularity through the way of rail material removal, so as to ensure that vehicle has better dynamic behavior running among the line after grinding. Metro line is characterized by small curvature radius, short transition section which has higher requirement on curve negotiating ability of rail grinder, especially when grinding is implemented. Table 1 shows nomenclature.

**Table 1:** Nomenclature.

$l_{c1x}$_,_ $l_{c1y}$_,_ $l_{c1z}$_,_ $l_{c2x}$_,_ $l_{c2y}$_,_$l_{c2z}$	*x*, *y*, *z* distance from mass center of grinding car to secondary suspension, to draw bar
$l_{b1x}$_,_ $l_{b1y}$_,_ $l_{b1z}$_,_ $l_{b2x}$_,_ $l_{b2y}$_,_ $l_{b2z}$_,_$l_{b3z}$	*x*, *y*, *z* distance from mass center of buggy frame to buggy suspension, to draw bar, to rotation center of cradle
$l_{d1z}$_,_ $l_{d1z}$_,_ $L_{g} (t)$_,_$l_{g}$	*z* distance from mass center of cradle to adjusting hydraulic cylinder, to rotation center, actual and nominal length from rotation center of cradle to grinding wheel
$x_{c}$_,_ $y_{c}$_,_ $z_{c}$_,_ $x_{b}$_,_ $y_{b}$_,_$z_{b}$	Mass center in *x*, *y*, *z* of grinding car, of buggy frame
$\phi_{c}$, $\beta_{c}$, $\psi_{c}$, $\phi_{b}$, $\beta_{b}$, $\psi_{b}$,$\phi_{d}$	Rotation angle of grinding car and of buggy in *x*, *y*, *z*, of cradle in *x*
${\mathbf{K}}_{sb1}$, ${\mathbf{C}}_{sb1}$, ${\mathbf{K}}_{g}$, ${\mathbf{C}}_{g}$, $K_{b}$,$C_{b}$	Vector stiffness and damping of draw bar, of grinding hydraulic cylinder, equivalent stiffness and damping of adjusting hydraulic cylinder
${\mathbf{F}}_{sb}$, ${\mathbf{F}}_{g}$,$T_{b}$	Suspension or traction vector force between grinding car and buggy, vector force between cradle and grinding wheel, torque of adjusting hydraulic cylinder
$R_{c}$, $R_{b}$	Curvature radius of grinding car center and of buggy center when running
${\mathbf{s}}_{c}$_,_ ${\mathbf{s}}_{b}$	Vector coordinate in grinding car and in buggy frame
$P_{1}$, $P_{2}$, $P_{3}$, $P_{4}$, $P_{a}$,$P_{b}$	Inlet, inner pressure of main valve, inlet pressure of pilot valve, pipe pressure of rod cavity, pressure of rodless cavity, of rod cavity
$m_{m}$, $m_{p}$,$m_{g}$	Mass of main valve piston, of pilot valve piston, of hydraulic cylinder piston
$A_{p}$, $A_{a}$, $A_{b}$, $A_{m}$,$A_{g}$	Action area of pilot valve, of rodless cavity, of rod cavity, of main valve, of rod cavity outlet
$x_{p}$, $x_{m}$, $z_{g}$,$z_{g0}$	Displacement of pilot valve, of main valve piston, of hydraulic cylinder piston, of initial position in hydraulic cylinder piston
$F_{0}$, $F_{e}$,$F_{h}$	Set, actual value of electromagnetism force, Hertzian force
$a$, $c$, $\rho$, $E$, $E^{*}$, $\delta$, $L$,$k$	Compensation coefficient, viscous damping coefficient, oil density, oil bulk Modulus, nominal elastic modulus, compression amount, extension of hydraulic cylinder, equivalent stiffness of hydraulic cylinder
$c_{d1}$, $c_{d2}$, $c_{d3}$, $c_{d4}$,$c{}_{r}$	Flow coefficient of pilot valve, of main valve port 1, of main valve port 2, of rodless cavity, of throttle valve
$\alpha_{p}$, $\alpha_{m}$, $k_{m}$,$l_{m}$	Half cone angle of pilot valve, flow angle of main valve, main valve stiffness, outlet width of main valve
$P$, $\omega$, $r_{1}$, $u$, $\mu$,$t$	Grinding pressure, rotation speed of grinding wheel, nominal radius of grinding wheel, control voltage, friction coefficient, time
$D_{p}$, $D_{m}$, $D_{r}$, $p$,$V$	Diameter of pilot valve, of main valve, of throttle valve chamber, pressure of hydraulic oil, volume of hydraulic oil
$Q_{1in}$, $Q_{1out}$, $Q_{2}$, $Q_{3in}$,$Q_{3out}$	Inlet, outlet flow of main valve, outlet flow of rod cavity, inlet, outlet flow of pilot valve
$V_{p}$, $V_{m}$, $V_{a}$, $V_{b}$, $V_{La}$,$V_{Lb}$	Volume of pilot valve and pipe, of main valve, of rodless cavity, of rod cavity, of rodless cavity pipe, of rod cavity pipe
$N_{0}$, $N_{t}$, $U$, $I$, $\varphi$,$\Delta$	Set power, actual power, motor voltage, motor current, power factor, compensation
$a_{0}$, $b_{0}$,$r_{0}$	Grinding width, grinding length, rail surface radius
$F_{y}$, $F_{z}$, $T_{y}$,$T_{z}$	Lateral, vertical force, lateral, vertical torque of grinding wheel

**Figure 1:**
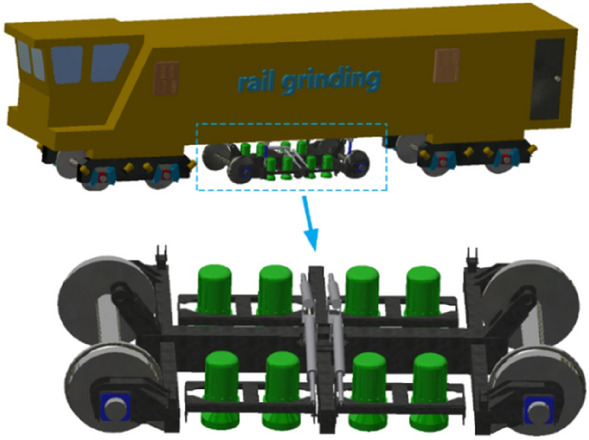
Rail grinder.

Rail grinder is composed of mechanical, hydraulic, and control system. Mechanical system includes grinding car, draw bar, buggy, motor, grinding wheel, etc. It is the executive part. Hydraulic system includes hydraulic cylinder, hydraulic pump, accumulator, pilot proportional reducing valve, one-way valve, directional valve, etc. It provides pressure for grinding. Control system includes current detection, signal processing, error correction, signal output, etc. It is the key component to maintain constant power grinding. The above systems are interrelated to form mechanical-electric-hydraulic coupling system of rail grinder. Figure 1 shows a typical rail grinder.

Grinding car interacting with rail through bogie frame, suspension and wheel, generates vibration in grinding process.

Buggy interacting with rail through suspension and guide wheel, also generates vibration. Structure parameters of the two are inconsistent, so dynamic vibrations are different. They affect each other through the action of draw bar. Buggy is connected with grinding wheel through hydraulic cylinder, and vibration of it will affect position of grinding wheel. Under action of hydraulic cylinder, grinding wheel exerts pressure on rail. Due to help of wheel rotation, pressure is transformed into grinding force, and rail material is cut off. Pressure can be approximately regarded as Hertzian force. Rail grinding is a sliding friction process, which can be approximated as friction in dynamic analysis and obeys Coulomb's theory. There is a moment arm between grinding force and center point, resulting in friction torque. According to reaction principle, rail will exert pressure, grinding force and friction torque on grinding wheel. For buggy, it is vertical force, lateral force and torque. These forces affect vibration state of buggy and grinding car Dynamic behavior of buggy and grinding car will affect grinding pressure also.

In order to ensure stability of grinding, grinding process needs to be controlled. Constant power grinding is generally adopted, which is accomplished by cooperation of hydraulic system and control system. Hydraulic system is equipped with pilot proportional reducing valve. Under action of it, pressure of hydraulic system is directly proportional to control voltage, but there is a lag. Control system obtains actual grinding power by detecting current of grinding motor. Pressure of hydraulic system is controlled by adjusting control voltage of that valve in real time through closed-loop feedback. When grinding power is low, system will keep pressure of rodless cavity greater than that of rod cavity, pushing rod of hydraulic cylinder out. Extension of hydraulic cylinder will produce greater Hertzian contact force and increase grinding power. And the opposite is the same. But stability control to grinding power is a dynamic process, not absolutely constant.

## Model of rail grinder

### Submodel of vehicle-track dynamic coupling

**Figure 2:**
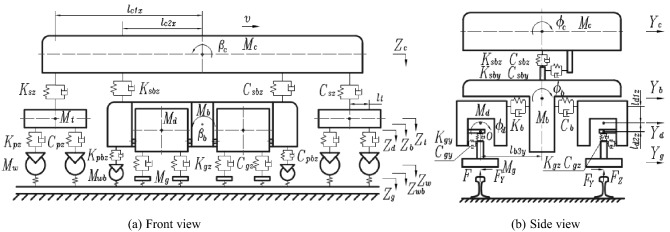
Vehicle track coupling model of rail grinder.

Under grinding mode, grinding car and buggy are connected through draw bar, and suspension hydraulic cylinder share no load at this time. Grinding wheel is in contact with rail, and the whole vehicle runs at a uniform speed. Different rail grinders have different structures, but they are all composed of grinding car and buggy, both of which confirm to Zhai-Sun model, that is vehicle track dynamic coupling model. There are many direction and pressure adjustment hydraulic cylinders in buggy. When hydraulic oil is charged, hydraulic cylinder can be equivalent to spring damping structure. Draw bar can also be equivalent to spring damping structure. The whole vehicle track coupling model of rail grinder is shown in Fig. 2, while point *O* is the rotation center of cradle.

Equivalent stiffness^20^ of charged hydraulic cylinder is:

1 $$ k = E\left( {\frac{{A_{a}^{2} }}{{V_{a} + V_{La} }} + \frac{{A_{b}^{2} }}{{V_{b} + V_{Lb} }}} \right), $$

Displacement of the two rigid bodies on both sides of draw bar are:

2 $$ {\mathbf{s}}_{ci} = \left[ \begin{gathered} x_{c} (t) \hfill \\ y_{c} (t) \hfill \\ z_{c} (t) \hfill \\ \end{gathered} \right] + \left[ {\begin{array}{*{20}c} 0 & {( - 1)^{i} \psi_{c} (t)} & {( - 1)^{i} \beta_{c} (t)} \\ {( - 1)^{i + 1} \psi_{c} (t)} & 0 & {( - 1)^{i + 1} \phi_{c} (t)} \\ {( - 1)^{i + 1} \beta_{c} (t)} & {( - 1)^{i} \phi_{c} (t)} & 0 \\ \end{array} } \right]\left[ {\begin{array}{*{20}c} {l_{c2x} } \\ {l_{c2y} } \\ {l_{c2z} } \\ \end{array} } \right], $$

3 $$ {\mathbf{s}}_{bi} = \left[ \begin{gathered} x_{b} (t) \hfill \\ y_{b} (t) \hfill \\ z_{b} (t) \hfill \\ \end{gathered} \right] + \left[ {\begin{array}{*{20}c} 0 & {( - 1)^{i} \psi_{b} (t)} & {( - 1)^{i} \beta_{b} (t)} \\ {( - 1)^{i + 1} \psi_{b} (t)} & 0 & {( - 1)^{i + 1} \phi_{b} (t)} \\ {( - 1)^{i + 1} \beta_{b} (t)} & {( - 1)^{i} \phi_{b} (t)} & 0 \\ \end{array} } \right]\left[ {\begin{array}{*{20}c} {l_{b2x} } \\ {l_{b2y} } \\ {l_{b2z} } \\ \end{array} } \right], $$

Considering coordinate system transformation, force of drawbar is:

4 $$ {\mathbf{F}}_{sbi} = {\mathbf{K}}_{sb1} ({\mathbf{s}}_{ci} - {\mathbf{s}}_{bi} + \Delta {\mathbf{s}}) + {\mathbf{C}}_{sb1} ({\dot{\mathbf{s}}}_{ci} - {\dot{\mathbf{s}}}_{bi} + \Delta {\dot{\mathbf{s}}}), $$

5 $$ {\text{While}},\quad \Delta {\mathbf{s}} = \left[ {\begin{array}{*{20}c} 0 \\ {\frac{{l_{c1x}^{2} }}{{2R_{c} }} - \frac{{l_{b1x}^{2} }}{{2R_{b} }}} \\ 0 \\ \end{array} } \right], $$

Torque at direction adjusting hydraulic cylinder is:

6 $$ T_{bi} = \left[ {K_{b} (\phi_{d} - \phi_{b} ) + C_{b} (\dot{\phi }_{d} - \dot{\phi }_{b} )} \right](l_{d1z} + l_{d2z} )^{2} , $$

Force at grinding hydraulic cylinder is:

7 $$ {\mathbf{F}}_{gi} = {\mathbf{K}}_{g} \left[ {\begin{array}{*{20}c} 0 \\ {(L_{g} (t) - l_{g} )\cos \phi_{d} } \\ {(L_{g} (t) - l_{g} )\sin \phi_{d} } \\ \end{array} } \right] + {\mathbf{C}}_{g} \left[ {\begin{array}{*{20}c} 0 \\ {\mathop {L{}_{g}(t)}\limits^{ \cdot } \cos \phi_{d} } \\ {\mathop {L{}_{g}(t)}\limits^{ \cdot } \sin \phi_{d} } \\ \end{array} } \right], $$

When calculating contact force between grinding wheel and rail, grinding wheel can be regarded as a plane and rail can be regarded as a curved surface. According to Carter theory^21^, width of contact band can be shown in Eq. (8) and contact pressure can be shown in Eq. (9).

8 $$ a_{0} = \left( {\frac{{4r_{0} P}}{{\pi E*b_{0} }}} \right)^{1/2} , $$

9 $$ P = \frac{{\pi E^{*} b_{0} }}{2}\delta , $$

Force between rail and grinding wheel is:

10 $$ \left\{ {\begin{array}{*{20}l} {F_{y} = \frac{{\pi E^{*} b_{0} }}{2}\delta \sin \phi_{d} } \hfill \\ {F_{z} = \frac{{\pi E^{*} b_{0} }}{2}\delta \cos \phi_{d} } \hfill \\ {T_{y} = \mu F_{y} r_{1} } \hfill \\ {T_{z} = \mu F_{z} r_{1} } \hfill \\ \end{array} } \right., $$

For force of other parts, please refer to reference^8^. Motion equations of all parts can be obtained through Newton's or Dalamber's theorem. At this time, rail grinder includes 20 rigid bodies: 1 grinding car, 1 buggy frame, 2 bogies, 4 wheelsets, 4 cradles and 8 grinding wheels. Cradle only has rotation DOF, and grinding wheel only has extension DOF. For other parts, 5 DOF are considered: transverse displacement, vertical displacement, rolling, pitching and yawing. So the whole rail grinder has 52 DOF.

### Submodel of hydraulic system

**Figure 3:**
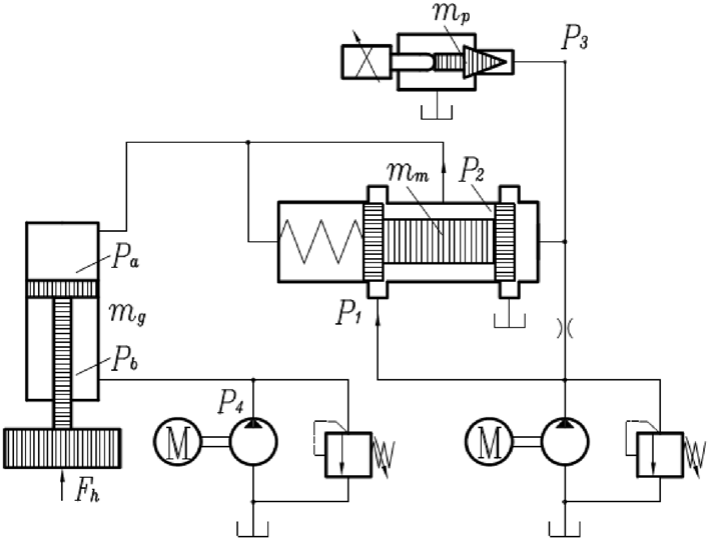
Hydraulic model.

Figure 3 shows the hydraulic model. Oil volume will affect oil pressure, and their relationship is shown in Eq. (11). Equations below can be inferred from Eq. (11). Equation (12) is the force balance of pilot valve. Equation (13) is the calculation method of electromagnetic force. Equation (14) is the formula for calculating pilot valve pressure through hydraulic flow. Equation (15) is the force balance formula of main valve. Equation (16) is the flow formula of main valve when oil is injected to hydraulic cylinder. Equation (17) is the flow formula of main valve when oil returns from hydraulic cylinder to oil tank. Equation (18) is the formula for calculating main valve pressure through hydraulic flow. Equation (19) is the force balance formula of piston rod inside hydraulic cylinder. Equation (20) is volume change formula of hydraulic cylinder in rodless cavity and rod cavity. Equation (21) is the calculation formula of extension amount for hydraulic cylinder.

Formation of hydraulic pressure^22^:

11 $$ dp = - E\frac{dV}{V}, $$

Pilot valve:

12 $$ P_{3} A_{P} = m_{p} \ddot{x}_{p} + c\dot{x}_{p} { + }F_{e} , $$

13 $$ F_{e} { = }F_{0} \left[ {1 + \frac{{a(N_{0} - N_{t} )}}{{N_{0} }}} \right], $$

14 $$ \left\{ \begin{gathered} Q_{3in} = c_{r} \frac{{\pi D_{r}^{2} }}{4}\sqrt {\frac{{2(P_{1} - P_{3} )}}{\rho }} \hfill \\ Q_{3out} = c_{d1} \pi D_{p} x_{p} \sin \alpha_{p} \sqrt {\frac{{2P_{3} }}{\rho }} \hfill \\ \mathop {P_{3} }\limits^{ \cdot } = - E\frac{{Q_{3out} - Q_{3in} }}{{V_{p} }} \hfill \\ \end{gathered} \right., $$

Main valve:

15 $$ P_{3} A_{m1} - {\text{2c}}_{d2}^{2} \pi D_{m} \cos \alpha_{m} x_{m} (P_{1} - P_{2} ) = m_{m} \ddot{x}_{m} + c\dot{x}_{m} { + }k_{m} x_{m} + P_{2} A_{m2} , $$

16 $$ {\text{Oil inject}}:\quad \left\{ {\begin{array}{*{20}l} {Q_{1in} = c_{d2} \pi D_{m} x_{m} \sqrt {\frac{{2(P_{1} - P_{2} )}}{\rho }} } \hfill \\ {Q_{1out} = c_{d3} \pi D_{m} l_{m} \sqrt {\frac{{2(P_{2} - P_{a} )}}{\rho }} } \hfill \\ \end{array} } \right., $$

17 $$ {\text{Oil return}}:\quad \left\{ {\begin{array}{*{20}l} {Q_{1in} = c_{d3} \pi D_{m} l_{m} \sqrt {\frac{{2(P_{a} - P_{2} )}}{\rho }} } \hfill \\ {Q_{1out} = c_{d2} \pi D_{m} x_{m} \sqrt {\frac{{2P_{2} }}{\rho }} } \hfill \\ \end{array} } \right., $$

18 $$ \mathop {P_{2} }\limits^{ \cdot } = - E\frac{{Q_{1out} - Q_{1in} }}{{V_{m} }}, $$

Hydraulic cylinder:

19 $$ \left\{ {\begin{array}{*{20}l} {P_{a} A_{a} = m_{g} \ddot{z}_{g} + c\dot{z}_{g} + P_{b} A_{b} { + }F_{h} } \hfill \\ {\mathop {P_{a} }\limits^{ \cdot } = - E\frac{{Q_{1} + \dot{z}_{g} A_{a} }}{{V_{a} + V_{La} }}} \hfill \\ {\dot{P}_{b} = - E\frac{{Q_{2} - \dot{z}_{g} A_{b} }}{{V_{b} }}} \hfill \\ {Q_{2} = c_{d4} A_{g} \sqrt {\frac{{2(P_{b} - P_{4} )}}{\rho }} } \hfill \\ {F_{h} = \frac{{\pi E^{*} b_{0} }}{2}\delta } \hfill \\ \end{array} ,} \right. $$

20 $$ \left\{ {\begin{array}{*{20}l} {\dot{V}_{a} = \dot{z}_{g} tA_{a} } \hfill \\ {\dot{V}_{b} = - \dot{z}_{g} tA_{b} } \hfill \\ \end{array} } \right., $$

21 $$ L = z_{g} - z_{g0} , $$

### Submodel of control system

**Figure 4:**
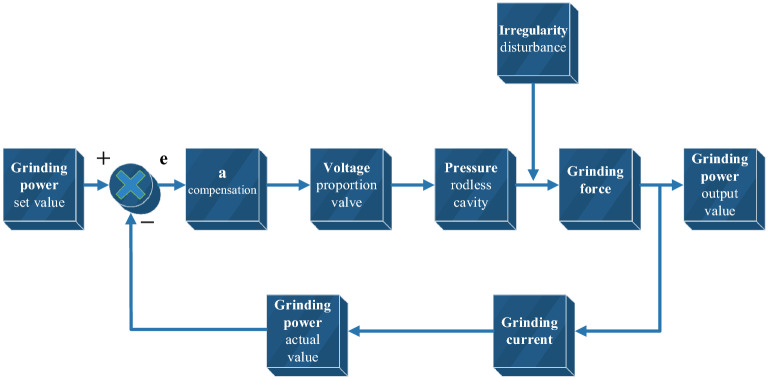
Control model.

Voltage *U* of grinding motor and power factor *φ* are approximately unchanged. A closed-loop feedback is set to compensate grinding power. In practice, there are many ways of deviation compensation, and this paper adopts linear compensation. Equation (22) is the calculation formula of grinding power. Equation (23) is the compensation amount. Figure 4 shows the control model.

22 $$ N_{t} = \sqrt 3 UI\cos \varphi = \mu P\omega r_{1} , $$

23 $$ \Delta = a\left( {\frac{{N_{0} - N_{t} }}{{N_{0} }}} \right), $$

Rail grinder runs on rail with uniform speed, and coordinates of upper hinge joint on grinding hydraulic cylinder are exported through vehicle-track model. Control model exports control voltage *u* to hydraulic model by detecting grinding motor current *I*. Hydraulic model receives control voltage *u* and adjusts pressure of hydraulic system. As a result, extension *L* of hydraulic cylinder changes under influence of pressure, and it is exported also. After receiving coordinates and extension amount *L*, program calculates compression amount *δ* by comparing with rail irregularity. Grinding force can be calculated from compression amount *δ*. Under action of grinding force, lateral force *F*_*y*_, vertical force *F*_*z*_, lateral torque *T*_*y*_, vertical torque *T*_*z*_ can be output to vehicle-track model, and Hertzian force *F*_*h*_ can be output to hydraulic system. In this iterative cycle, curve negotiating ability of rail grinder in the whole line can be obtained.

## System simulation

**Figure 5:**
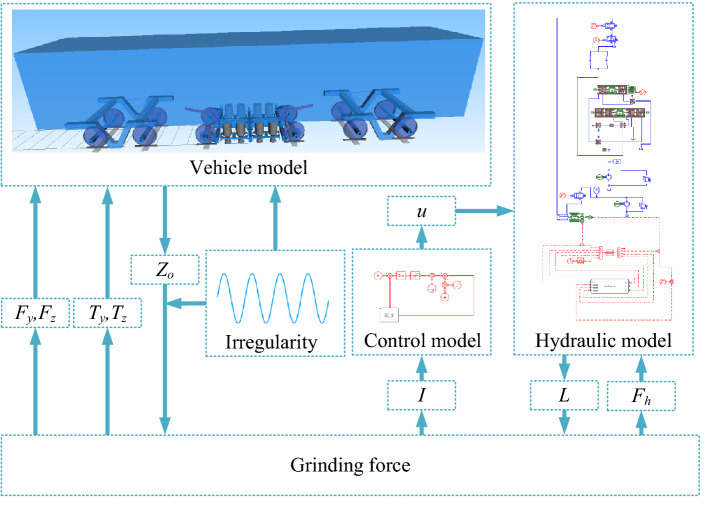
Mechanical-electric-hydraulic coupling model.

Rail grinder involves three parts: vehicle-track system, hydraulic system and control system, which need to be modeled separately. Through interface program, the three can transfer parameters and realize interaction. Figure 5 shows the mechanical-electric-hydraulic coupling model.

Taking Tianjin Metro Line 1 as an example, rail corrugation on upper side rail of curve section is serious. It is mainly composed of long wave, and the amplitude can reach mm level^23^. The line selected in this paper is shown in Table 2, and standard sinusoidal excitation is applied as vertical irregularity of rail to imitate rail corrugation, as shown in Eq. (24). In this paper, we simulate grinding mode of rail top surface trimmed, that is, set angle of cradle is 0°. According to actual rail damage, method of outer rail one-side grinding is adopted. Curve negotiating ability can be analyzed by simulation.

24 $$ y = A\sin \left( {\frac{2\pi x}{{L_{0} }}} \right),\quad A = 0.5\,{\text{mm,}}\quad L_{0} = 5\,{\text{m,}} $$

**Table 2:** Line.

Item	Parameter	Value
1	Straight length at both ends	45 m
2	Transition curve length at both ends	55 m
3	Curve radius	800 m
4	Curve length	60 m
5	Superelevation	95 mm
6	Travelling speed	16 kh/h

During rail grinding, change of grinding parameters will affect curve negotiating ability of rail grinder. In order to evaluate influence of those parameters, 4 cases are set. At this moment, we focus on the peak value. Table 3 shows grinding parameters and cases. According to function of rail grinder, evaluation and test method of its dynamic behavior shall comply with GB/T 17426-1998. All dynamic behavior talking inside this paper are based on this standard. Unspecified dynamic characteristics in this paper refer to that of buggy. “with grinding” refers to dynamic characteristics of buggy in grinding state, and “without grinding” refers to dynamic characteristics of buggy not in grinding state.

**Table 3:** Grinding parameters.

Case	Irregularity amplitude (mm)	Irregularity wavelength (m)	Line curvature radius (m)	Number of grinding wheels at work
Typical	0.5	5	800	4
1	0.5, 0.6, 0.7, 0.8, 0.9, 1	5	800	4
2	0.5	1, 2, 3, 4, 5	800	4
3	0.5	5	300, 400, 500, 600, 700, 800	4
4	0.5	5	800	1, 2, 3, 4

## Analysis of grinding power and curve negotiating ability

### Typical case

**Figure 6:**
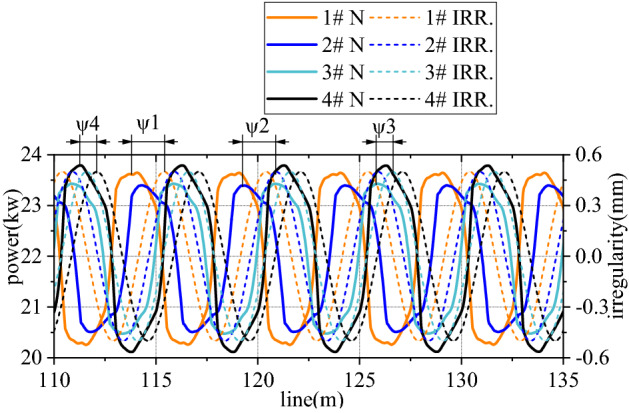
Position and grinding power.

**Figure 7:**
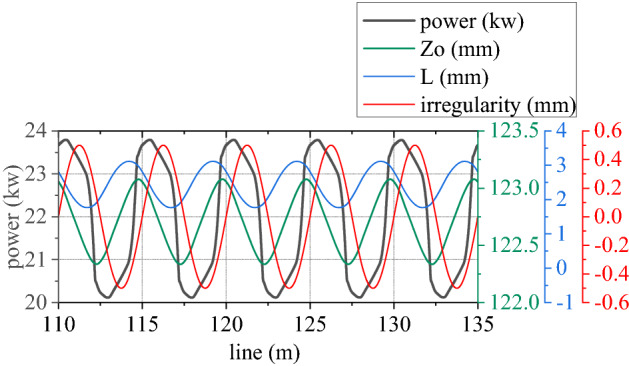
Grinding power and influence factor.

**Figure 8:**
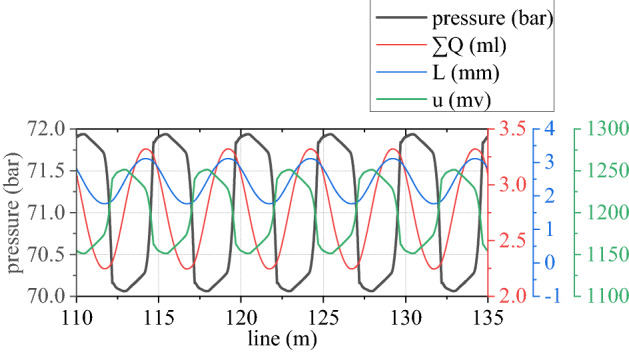
Pressure of rodless cavity and influencing factors.

**Figure 9:**
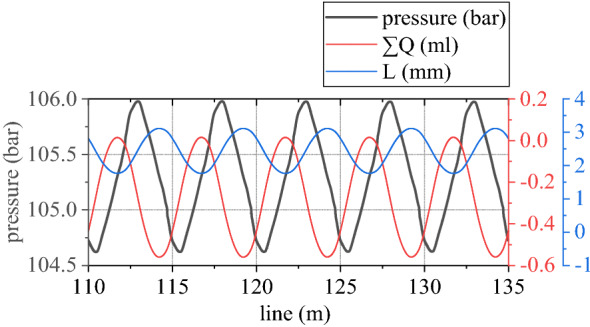
Pressure of rod cavity and influencing factors.

**Figure 10:**
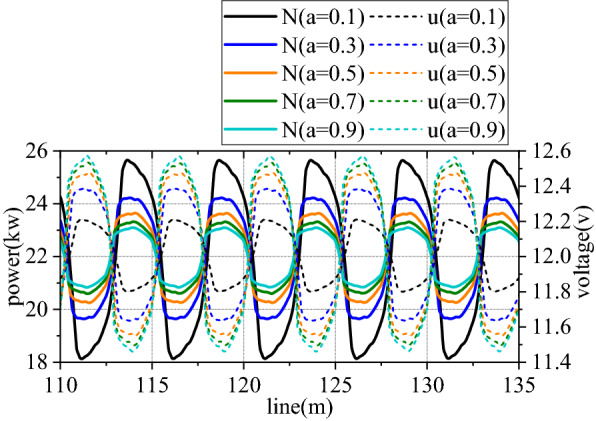
Control parameter and grinding power.

**Figure 11:**
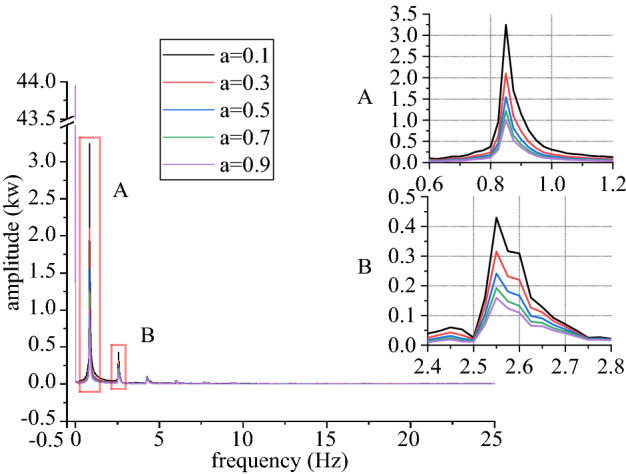
Spectrum characteristics of grinding power.

Figures 6, 7, 8, 9, 10 and 11 shows mechanical-electric-hydraulic coupling relationship of rail grinder under typical case. Phase difference for grinding wheels at different initial positions are different, and fluctuation amplitude of grinding power are also different. Phase difference is caused by non-coincidence of grinding wheel and buggy wheel, and fluctuation amplitude of grinding power is caused by pitching motion of buggy frame. For constant power grinding, phase difference and power fluctuation are unfavorable factors.

There is hydraulic pressure in grinding hydraulic cylinder and Hertz force in the grinding wheel. This structure can be approximately regarded as a series spring. Length of this spring is the value that vertical coordinate *Z*_*o*_ of cradle minus extension length *L* of hydraulic cylinder and rail irregularity. Grinding power is proportional to difference between spring length and spring nominal length. If grinding power is regarded as output, *Z*_*o*_ and rail irregularity can be regarded as passive input, while *L* can be regarded as positive input. So grinding power can be controlled by *L*.

Pressure of rodless cavity in grinding hydraulic cylinder is directly proportional to difference between its accumulated flow $\sum Q$ and $A_{a} L$. Pressure of rod cavity is directly proportional to sum between its accumulated flow $\sum Q$ and $A_{b} L$. If *L* is regarded as passive input temporarily, $\sum Q$ of rodless cavity can be regarded as positive input, pressure of rodless cavity can be regarded as positive output, $\sum Q$ of rod cavity can be regarded as passive input, and pressure of rod cavity can be regarded as passive output. Output *L* is controlled by pressure difference between the two cavities. Control voltage is proportional to pressure of rodless cavity, which is realized by changing flow $\sum Q$ of rodless cavity. As can be seen from Fig. 8, control voltage is reversed with pressure of rodless cavity to achieve grinding power fluctuation control.

For rail surface grinding, grinding power is directly proportional to vertical force *F*_*z*_ and torque *T*_*z*_ from rail to grinding wheel. Transverse force *F*_*y*_ and torque *T*_*y*_ from rail to grinding wheel can be ignored. Curve negotiating ability can be obtained by inputting *F*_*z*_ and *T*_*z*_ into the vehicle track model.

**Figure 12:**
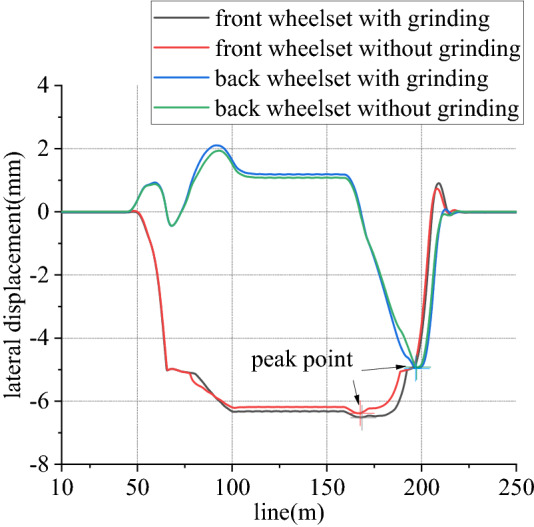
Lateral displacement of wheelsets (typical case).

**Figure 13:**
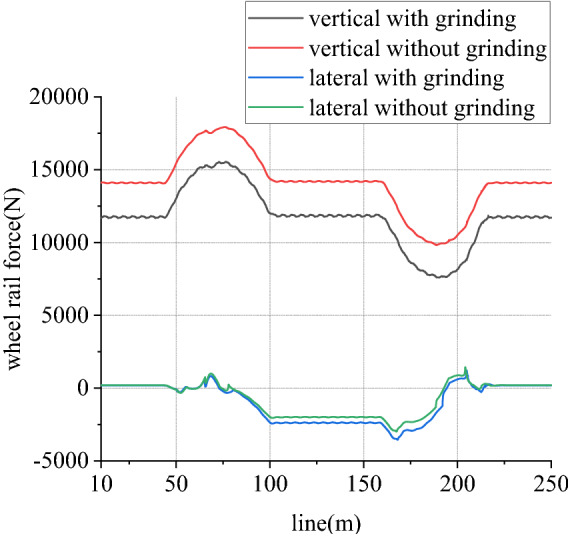
Wheel rail force of left wheel in front wheelset (typical case).

**Figure 14:**
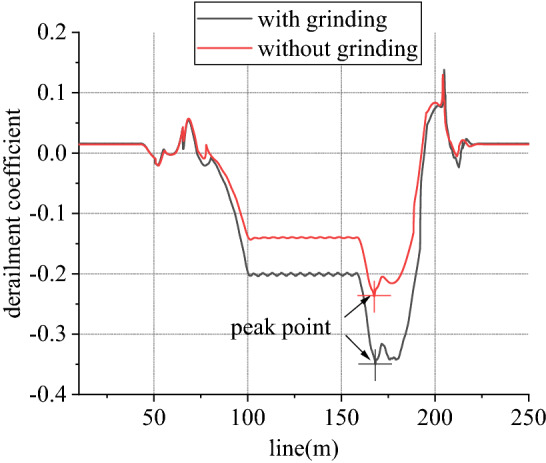
Derailment coefficient of left wheel in front wheelset (typical case).

**Figure 15:**
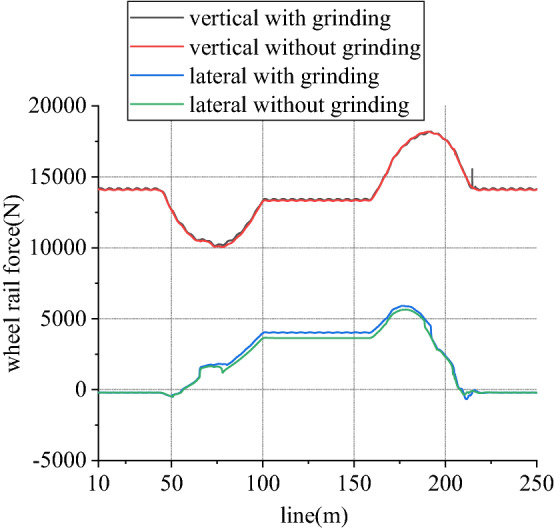
Wheel rail force of right wheel in front wheelset (typical case).

**Figure 16:**
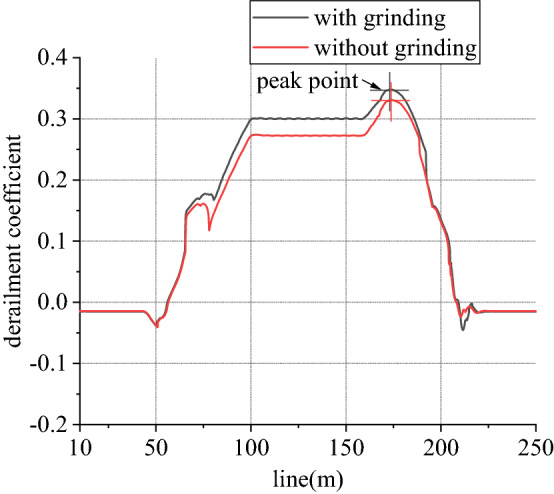
Derailment coefficient of right wheel in front wheelset (typical case).

**Figure 17:**
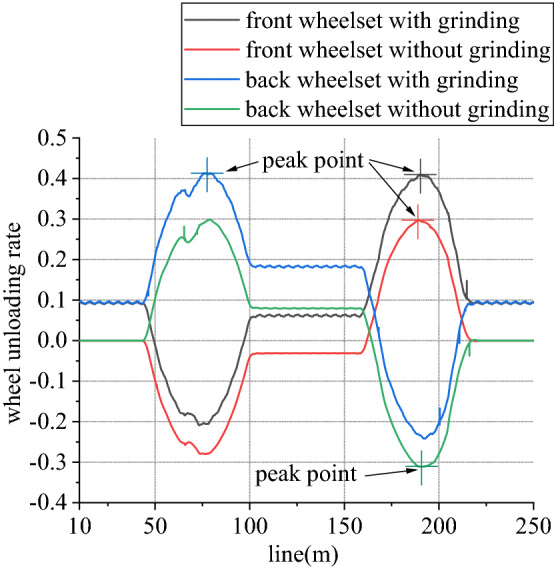
Unloading ratio (typical case).

Figures 12, 13, 14, 15, 16 and 17 shows curve negotiating ability of buggy under typical case.

Because wheelbase of buggy is large, in curve line, transverse displacement of front wheelset is negative and that of back wheelset is positive. It can be seen from Fig. 12, in curve line transverse displacements of both wheelsets increase, but it is not obvious. This is caused by vertical grinding force exerted on one-side way and grinding torque in *z* direction. And, torque in *x* direction from on-side load produced more contributes.

When work, speed of rail grinder is below 20 km/h, while that of metro vehicle can reach 80 km/h. In existing line, to rail grinder, superelevation is always surplus, so transverse force is positive. Due to existence of vertical grinding pressure, it can share and reduce wheel rail vertical force. Considering one-side load of grinding vertical force, load reduction effect of left wheel is greater than that of right wheel. From Figs. 13 and 14, for left wheel of front wheelset, lateral force increases, vertical force decreases, so derailment coefficient increases. From Figs. 15 and 16, for right wheel of front wheelset, lateral force increases, vertical force changes little, so derailment coefficient still increases. Maximum value of derailment coefficient in left wheel increases by about 47.1% and that in right wheel increases by 5.5%, compared to state without grinding.

As it can be seen from Fig. 17, for front wheelset, when enter transition section, vertical force of left wheel increases and that of right wheel decreases. When leave transition section, it is just opposite. And the opposite is true for back wheelset. When influence of vertical grinding pressure from one-side load is exerted, compared to state without grinding, vertical force of left wheel reduces, and that of right wheel is basically unchanged. For the whole line, maximum value of unloading ratio increases. That of front wheelset increases by about 40.2%, and back wheelset increases by 34.2%, compared to state without grinding.

### Influence of grinding parameters

For studying influence of different grinding parameters, this paper extracts peak points to analyze.

**Figure 18:**
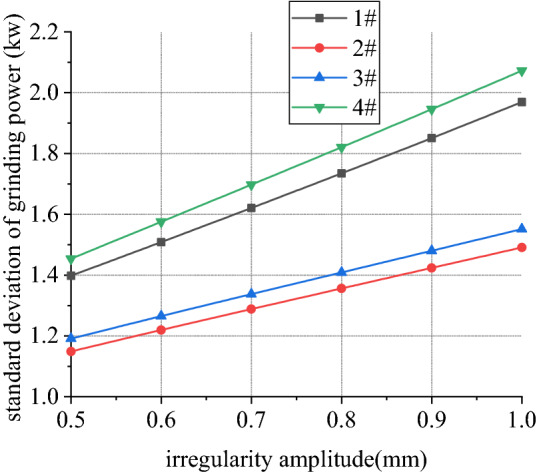
Amplitude and grinding power (case 1).

**Figure 19:**
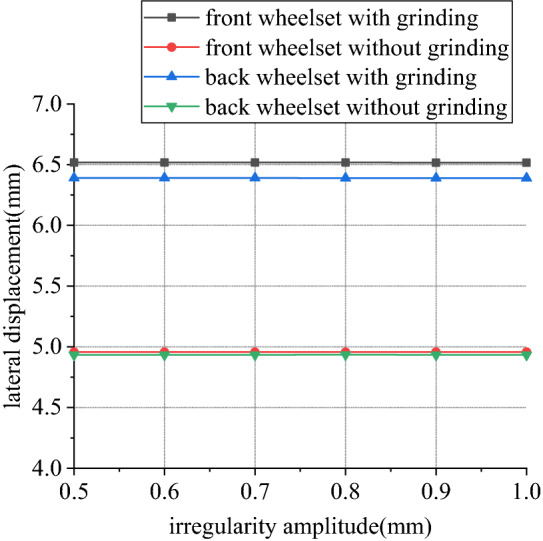
Amplitude and lateral displacement (case 1).

**Figure 20:**
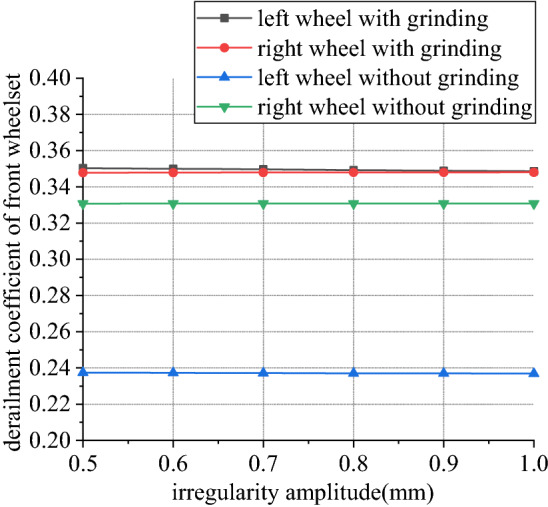
Amplitude and derailment coefficient (case 1).

**Figure 21:**
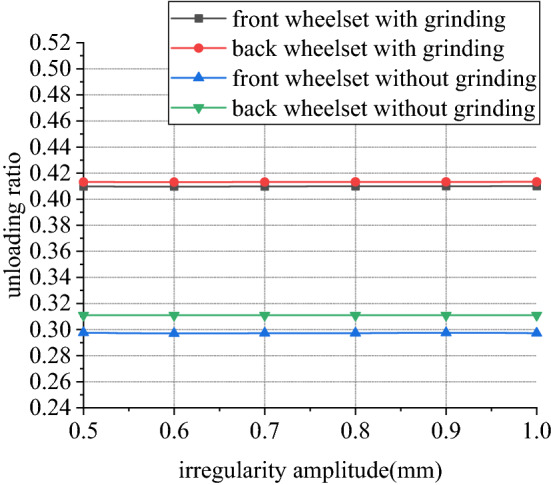
Amplitude and unloading ratio (case 1).

Figures 18, 19, 20 and 21 shows curve negotiating ability of buggy in case 1. As irregularity amplitude increases, fluctuation of grinding power increases. There is little change in wheelset lateral displacement, derailment coefficient and unloading ratio. Increase of rail irregularity increases fluctuation of Hertzian force between grinding wheel and rail, so fluctuation amplitude of grinding power increases. Uneven grinding will worsen grinding effect, which is an adverse effect. Within the given amplitude range of irregularity, fluctuation of Hertzian force is not enough to affect curve negotiating ability of buggy. Under the influence of primary longitudinal stiffness, lateral displacement of front wheelset is obviously greater than that of back wheelset. Since buggy has the following two characteristics:1.Compared with ordinary train, travelling speed is slow. When pass curve section, the superelevation of line is always too high, which is disadvantageous to curve negotiation ability. 2. 8 grinding wheels need to be installed on buggy. Wheelbase of buggy is generally large to reserve space for grinding wheels, which is also a disadvantageous factor. The above characteristics further increase the difference in lateral displacement between front and back wheelset.

**Figure 22:**
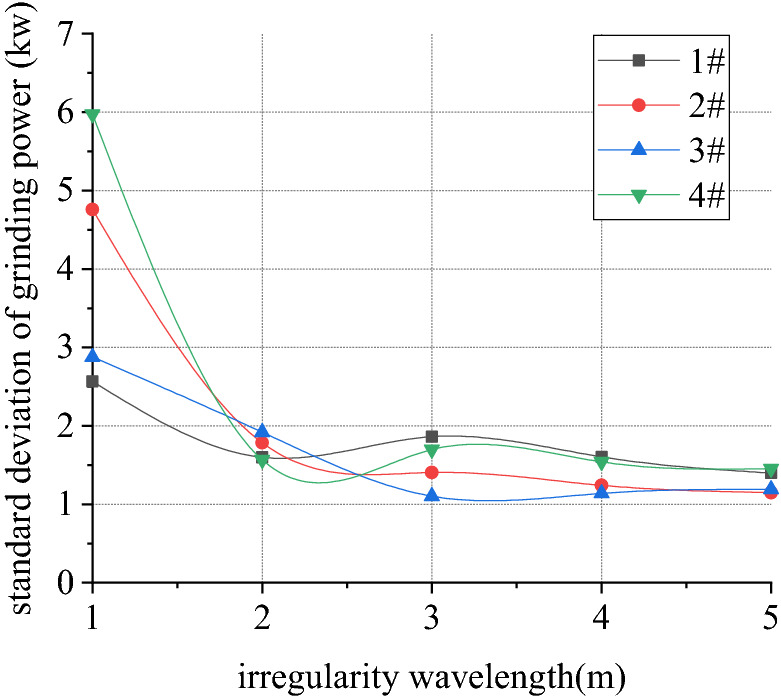
Wavelength and grinding power (case 2).

**Figure 23:**
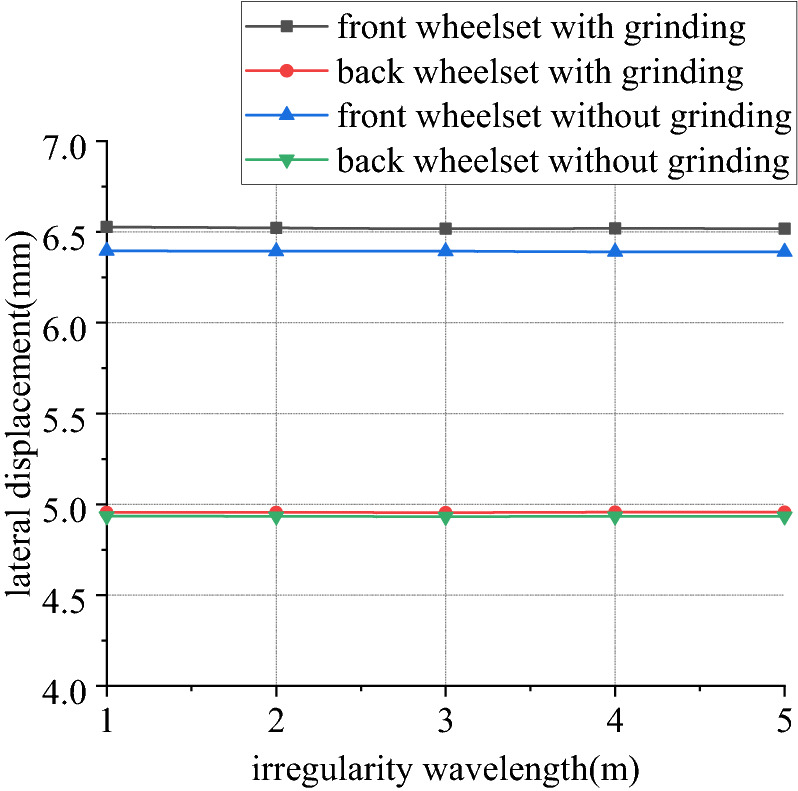
Wavelength and lateral displacement (case 2).

**Figure 24:**
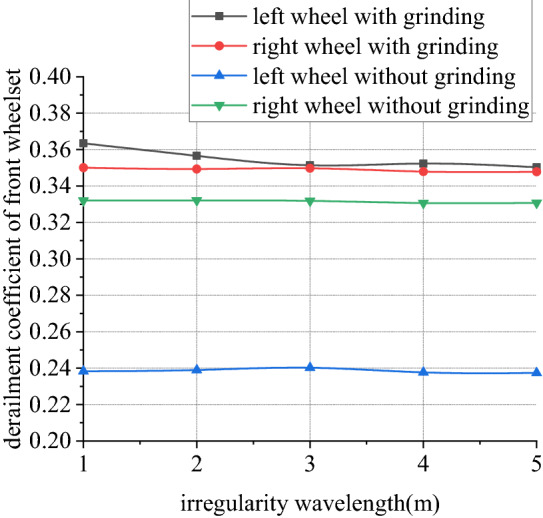
Wavelength and derailment coefficient (case 2).

**Figure 25:**
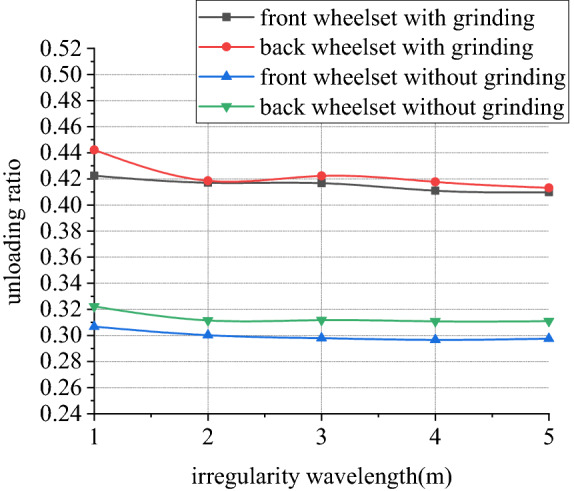
Wavelength and unloading ratio (case 2).

Figures 22, 23, 24 and 25 shows curve negotiating ability of buggy in case 2. As irregularity wavelength reduces, fluctuation of grinding power increases obviously. There is little change in wheelset lateral displacement. Derailment coefficient generally changes little, and that of left wheel in front wheelset increases slightly. Unloading ratio increases, but it is not obvious. Decrease of the wavelength of rail irregularity, increases fluctuation frequency of Hertzian force between grinding wheel and rail, and increases vibration amplitude of buggy. Finally, fluctuation amplitude of grinding power increases significantly and grinding effect is deteriorated. Within the given wavelength range of irregularity, fluctuation of Hertzian force is not enough to affect curve negotiating ability of buggy.

**Figure 26:**
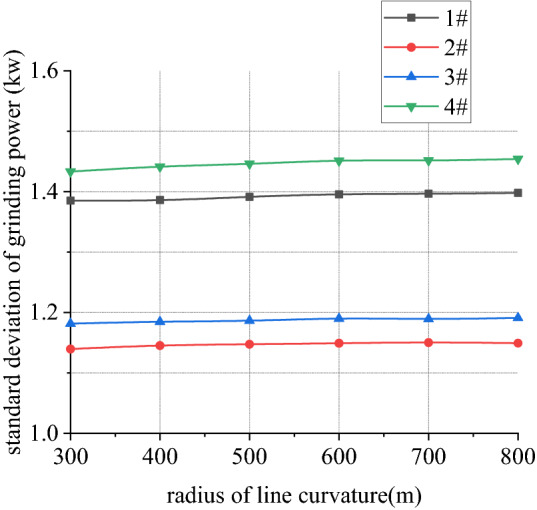
Curvature radius and grinding power (case 3).

**Figure 27:**
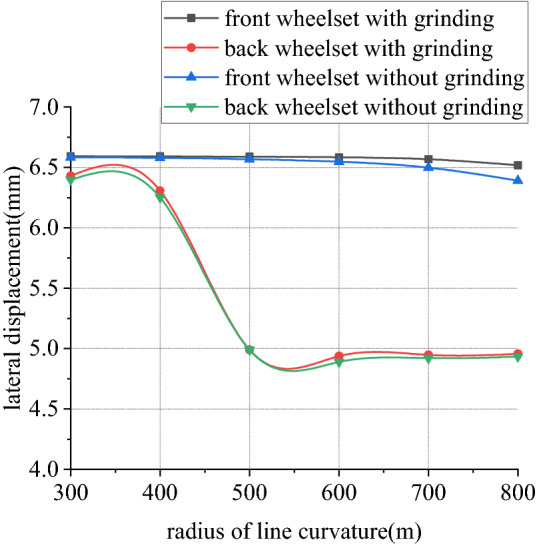
Curvature radius and lateral displacement (case 3).

**Figure 28:**
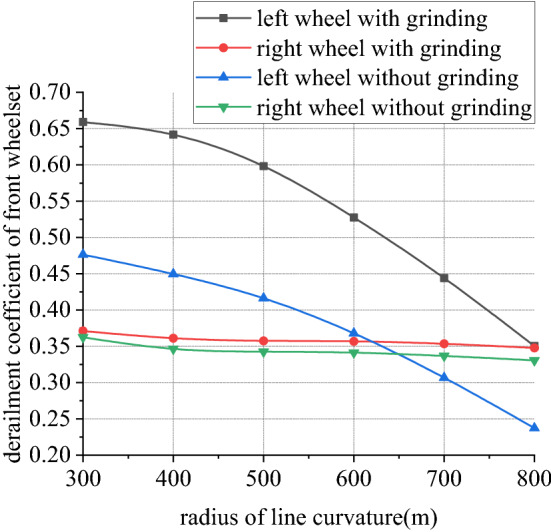
Curvature radius and derailment coefficient (case 3).

**Figure 29:**
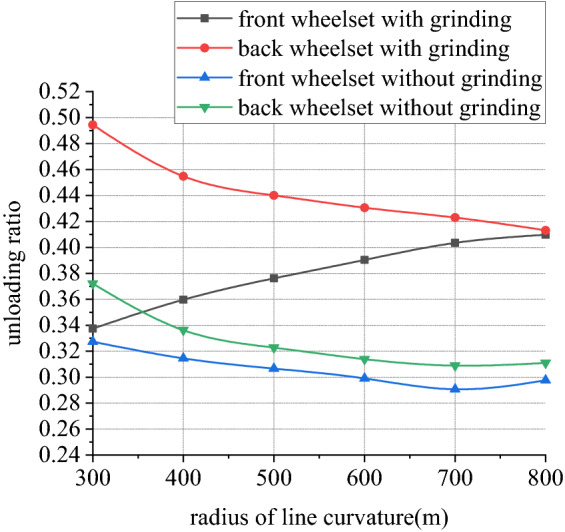
Curvature radius and unloading ratio (case 3).

Figures 26, 27, 28 and 29 shows curve negotiating ability of buggy in case 3. As curvature radius decreases, fluctuation power decreases slightly. Transverse displacement of wheelset increases, and that of back wheelset is very obvious. Derailment coefficient increases, and that of left wheel in front wheelset increases very obviously. Unloading ratio is differentiated. Without grinding, unloading ratio of wheelset increases with decrease of curvature radius. With grinding, that of front wheelset decreases and back wheelset increases when curvature radius decreases. This is mainly due to influence of one-side grinding that vertical force exerted on transition sections. Dynamic behavior when enter transition section and leave are different. With decrease of curve curvature radius, curve negotiating ability of states with grinding and without grinding both decrease, but decline of the state with grinding is greater. Grinding process deteriorates curve negotiating ability.

**Figure 30:**
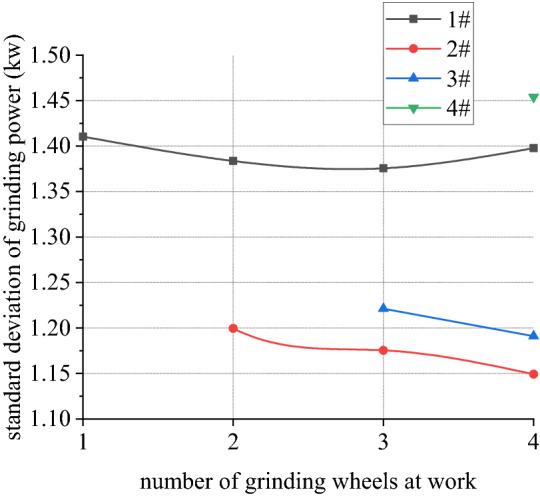
Grinding wheels and grinding power (case 4).

**Figure 31:**
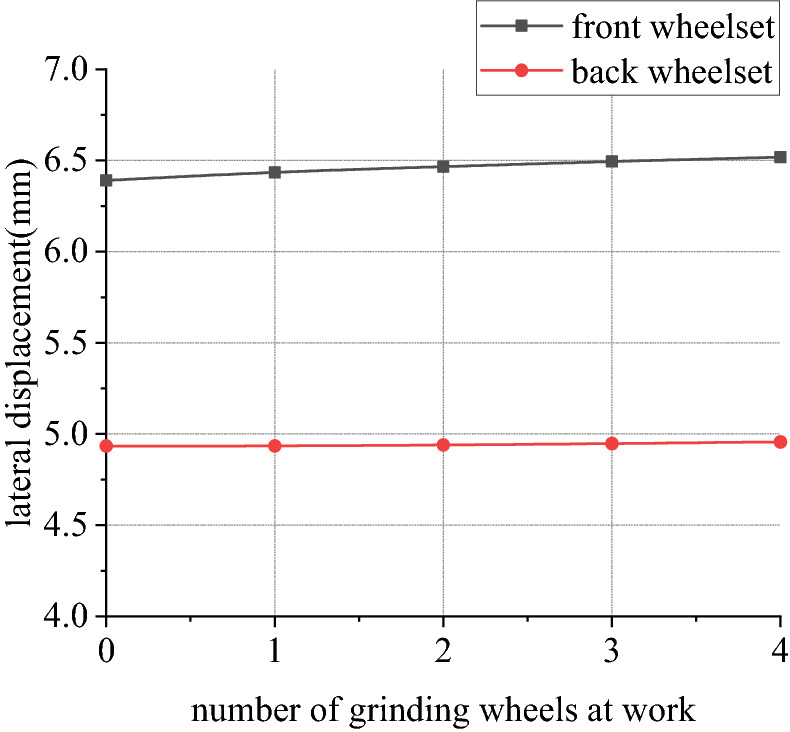
Grinding wheels and lateral displacement (case 4).

**Figure 32:**
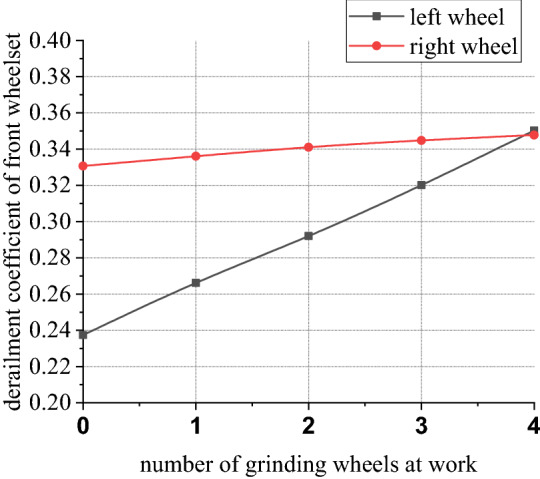
Grinding wheels and derailment coefficient (case 4).

**Figure 33:**
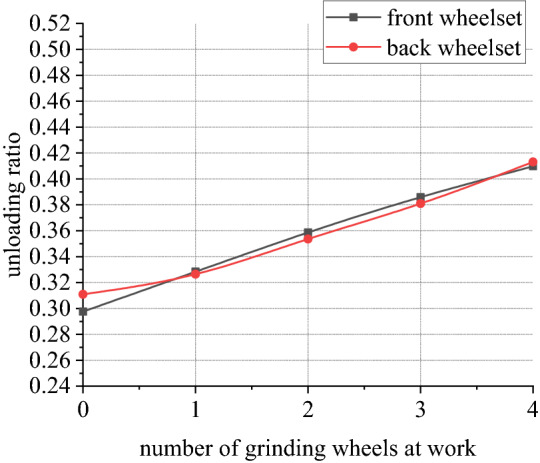
Grinding wheels and unloading ratio (case 4).

Figures 30, 31, 32 and 33 shows curve negotiating ability of buggy in case 4. As number of grinding wheels at work increases, fluctuation of grinding power has little changes which is only related to grinding wheel position. It generally conforms to behavior of independent grinding. While number of grinding wheels at work increases, lateral displacement of wheelset increases, and increase range of front wheelset is more obvious than that of back wheelset. Derailment coefficient increases, and that of left wheel in front wheelset is more obvious than that of right wheel in front wheelset. Unloading ratio also increases. Increasing number of grinding wheels at work will significantly increase total grinding pressure, which will inevitably affect curve negotiating ability of rail grinder.

## Conclusion

Dynamic behavior of rail grinder is due to vehicle-track coupling relationship built on mechanical-electric-hydraulic coupling. Modeling and simulation of the whole system under outer rail one-side grinding are exerted in this paper. The result is shown as below.

In typical case, transverse displacement of wheelsets in buggy increases. To left wheel of front wheelset, lateral force increases and vertical force decreases, so derailment coefficient increases. To right wheel of front wheelset, lateral force increases and vertical force changes little, and derailment coefficient still increases. Unloading ratio of wheelsets increases. This is mainly caused by vertical grinding force exerted on one-side way making wheel-track contact relationship changed.

In other 4 cases, irregularity amplitude and wavelength only affect fluctuation of grinding power. Increase of irregularity amplitude and decrease of irregularity wavelength will aggravate fluctuation of grinding power, but they have little effect on curve negotiating ability of rail grinder. Reduction of curvature radius has little effect on fluctuation of grinding power, but it increases transverse displacement of wheelsets and derailment coefficient of wheels in front wheelset, reduces unloading rate of front wheelset and increases unloading rate of back wheelset. Curve negotiating ability under the state with grinding and without grinding are all decreased but declined range of state with grinding is greater. Increase of grinding wheels at work will increase lateral displacement of wheelsets, derailment coefficient of wheels in the front wheelsets, and unloading rate of wheelsets.

In short, rail grinding has an impact on dynamic behavior of rail grinder, which will significantly deteriorate its curve negotiating ability. And curve negotiating ability in grinding state is still inside the range of standard.

## Data Availability

The datasets used and/or analysed during the current study available from the corresponding author on reasonable request.
